# The role of serum growth hormone and insulin-like growth factor-1 in adult humans brain morphology

**DOI:** 10.18632/aging.102688

**Published:** 2020-01-22

**Authors:** Taoyang Yuan, Jianyou Ying, Lu Jin, Chuzhong Li, Songbai Gui, Zhenye Li, Rui Wang, Zhentao Zuo, Yazhuo Zhang

**Affiliations:** 1 Beijing Neurosurgical Institute, Capital Medical University, Beijing, China; 2 Department of Neurosurgery, Beijing Tiantan Hospital, Capital Medical University, Beijing, China; 3 State Key Laboratory of Brain and Cognitive Science, Institute of Biophysics, Chinese Academy of Sciences, Beijing, China; 4 CAS Center for Excellence in Brain Science and Intelligence Technology, Chinese Academy of Sciences, Beijing, China; 5 Sino-Danish College, University of Chinese Academy of Sciences, Beijing, China; 6 Beijing Institute for Brain Disorders, Brain Tumour Center, China National Clinical Research Center for Neurological Diseases, Key Laboratory of Central Nervous System Injury Research, Beijing, China

**Keywords:** growth hormone (GH), insulin-like growth factor-1 (IGF-1), brain structure, structural magnetic resonance imaging analysis

## Abstract

Growth hormone (GH) and its anabolic mediator, insulin-like growth factor-1 (IGF-1), have a critical role in the central nervous system. However, their detailed roles in the adult human brain are not clear. In this study, structural MRIs of 48 patients with GH-secreting pituitary adenoma (GH-PA), 48 sex- and age-matched clinical Non-Functional pituitary adenoma patients (NonFun-PA) and healthy controls (HCs) were assessed using voxel-based morphometry (VBM) and region-based morphometry (RBM). Correlation analyses helped determine the relationships between serum hormone levels and brain structure. The whole-brain gray matter volume (GMV) and white matter volume (WMV) significantly increased at the expense of cerebrospinal fluid volume (CSFV) in GH-PA (Bonferroni corrected, p<0.01). The increase in GMV and reduction in CSFV were significantly correlated with serum GH/IGF-1 levels (p<0.05). VBM showed significant correlations of the GMV/WMV alteration pattern between GH-PA vs HCs and GH-PA vs NonFun-PA and widespread bilateral clusters of significantly increased GMV and WMV in GH-PA (pFDR<0.05). RBM showed obviously increased GMV/WMV in 54 of 68 brain regions (p<0.05) in GH-PA compared to HCs. Our results provide imaging evidence that serum GH/IGF-1 contributes to brain growth, which may be a potential treatment option for neurodegenerative disorders and brain injury in humans.

## INTRODUCTION

Growth hormone (GH) is secreted by somatotrophs within the anterior pituitary gland and leads to the production of insulin-like growth factor (IGF-1) in the liver [1]. GH and IGF-1 signaling are critical for the proper development and function of many major organ systems by regulating the growth of somatic cells, especially in early development and during adolescence [2, 3]. Previous animal studies reported robust expression of GH and IGF-1 receptors in the central nervous system (CNS), including the cerebral cortex, thalamus, hypothalamus, cerebellum, brainstem, and hippocampus [4, 5]. Meanwhile, GH and IGF-1 in circulation have been shown to cross the blood-brain barrier into the brain tissue and cerebrospinal fluid (CSF) to activate GH/IGF-1 receptors [3, 6–8]. The potential roles of GH and IGF-1 in brain growth, development, neurogenesis, and neuroprotection, as well as their functional roles in behavior, cognition and neurotransmission, have been investigated in vivo and in vitro [9–12]. For instance, GH or IGF-1 gene knock-out mice, compared to control animals, exhibited CNS deficits including reduced brain size, loss of myelination and specific parvalbumin-containing neurons, and cognitive impairments [13, 14]. Long-term GH/IGF-1 replacement has been shown to improve learning and memory in aged rats [15–17]. In animal models of neurodegenerative diseases, GH/IGF-1 administration improved cognition function [18]. Furthermore, exogenous GH/IGF-1 administration facilitated the proliferation, differentiation, survival, and migration of neural stem/precursor cells after brain injury in animals [19, 20]. In humans, some studies have suggested that GH/IGF-1 treatment can contribute to brain repair and cognitive function improvement in patients suffering from traumatic brain injury, GH deficiency, and neurodegenerative diseases [21–24]. The potential mechanism by which GH/IGF-1 affects the brain is through activating GH/IGF-1 receptors in the brain, including these on neurons, stem cells, and glial cells, and through the local induction and release of a number of neurotrophic factors, such as vascular endothelial growth factor, epidermal growth factor, and brain-derived neurotrophic factor [25–27]. However, the detailed effects of GH/IGF-1 on brain structure and the relationship between brain structure and serum levels of GH/IGF-1 have not been investigated in humans.

GH-secreting pituitary adenoma (GH-PA) is characterized by excess GH production and a concomitant increase in IGF-1 levels, leading to enlargement of the extremities, face, and soft tissues, macroglossia and dental changes in patients [28]. The excess circulating GH/IGF-1 levels in patients with GH-PA provides a specific model to characterize the effect of excess circulating GH/IGF-1 on brain gray matter (GM) and white matter (WM) in humans.

In this study, 48 patients with GH-PA, 48 age- and sex-matched clinical non-functional pituitary adenoma (NonFun-PA) patients, and 48 age- and sex-matched healthy controls (HCs) were recruited. We hypothesized that excess circulating GH/IGF-1 levels lead to volume increases in GM and WM, and are significantly related to brain structure volume.

## RESULTS

### Demographic and clinical factors

Forty-eight GH-PA patients, forty-eight age- and sex-matched NonFun-PA patients and forty-eight age- and sex-matched HCs were included. All patients in the GH-PA group underwent surgical treatment via the transsphenoidal approach, and had postoperative pathological diagnosis of pituitary GH-secreting adenoma. The demographic and clinical features are shown in Table 1.

**Table 1:** Demographic and clinical characteristics of the participants.

**Characteristic**	**GH-PA (n = 48)**	**NonFun-PA (n = 48)**	**Control (n = 48)**	**P-value**
Age (years)	43.3 (20-68)	43.3 (20-68)	43.3 (20-68)	1.000^a^
Sex (M/F)	24/24	24/24	24/24	1.000^b^
Serum GH (ng/ml)	14 (0.3-40)	0.3 (0.1-2.4)	NA	< 0.001^c^
Serum IGF-1 (ng/ml)	748 (199-1415)	161 (58-437)	NA	< 0.001^c^
Serum COR (ng/ml)	108 (40-209)	103 (10-228)	NA	0.5489^c^
Serum PRL (ng/ml)	11 (0.5-66.2)	14 (0.5-54.5)	NA	0.1698^c^
Serum LH (mIU/ml)	5.6 (0.6-32.6)	6.4 (0.1-43.3)	NA	0.6311^c^
Serum FSH (mIU/ml)	12 (0.8-77.9)	16 (1.2-145)	NA	0.3831^c^
Serum E2 (pg/ml)	50 (20-176)	50 (20-381)	NA	0.9471^c^
Serum P4 (ng/ml)	0.7 (0.2-7.9)	0.4 (0.2-4.4)	NA	0.2322^c^
Serum T (ng/ml)	0.9 (0.2-4.2)	1 (0.2-5.4)	NA	0.6640^c^
Serum TSH (uTu/ml)	1.3 (0.2-2.9)	1.5 (0.2-4.7)	NA	0.2105^c^
Therapy	surgery	NA	NA	NA
Pathology	GH (+)	NA	NA	NA

^a^ One-way ANOVA; ^b^ Fisher’s exact test, two-sided; ^c^ Student’s t-test between GH-PA and NonFun-PA, two-sided. GH, growth hormone; IGF-1, insulin-like growth factor-1; GH-PA, GH-secreting pituitary adenoma; NonFun-PA, non-function pituitary adenoma patients; COR, cortisol; PRL, prolactin; LH, luteinizing hormone; FSH, follicle-stimulating hormone; E2, estradiol; P4, progesterone; T, testosterone; TSH, thyroid stimulating hormone.

### The effect of excess GH/IGF-1 production on global brain volume

**Figure 1:**
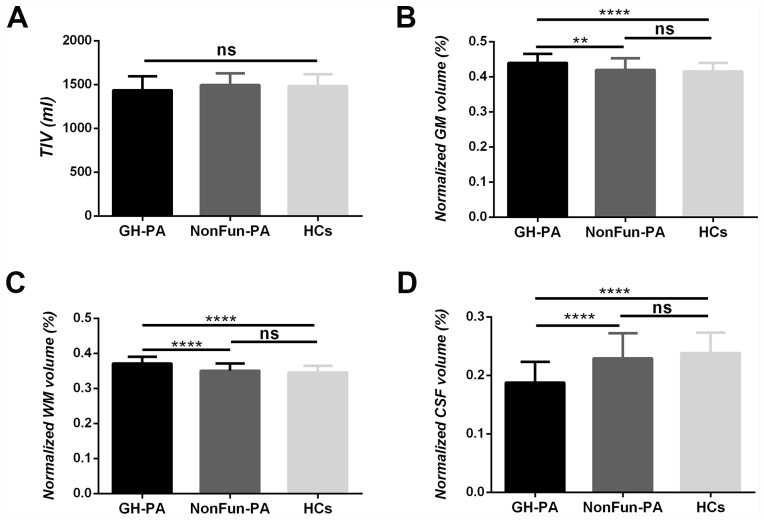
**Brain tissue volume changes in patients with GH-PA compared to NonFun-PA and HCs.** Statistical analysis of total intracranial volume (TIV) (**A**), normalized GM volume (nGMV) (**B**), normalized WM volume (nGWV) (**C**), and normalized CSF volume (nCSFV) (**D**) among three groups. Significance was determined by one-way ANOVA with Bonferroni's multiple comparisons test. ns represents no significant difference, ** p < 0.01, **** p < 0.0001.

Whole-brain analyses provided reliable evidence about whether excess GH/IGF-1 production impacted the entire brain structure, including the TIV, nGMV, nWMV, and nCSFV. As shown in Figure 1A, in our direct comparisons among the three groups, there were no significant differences in TIV. Next, we examined whether the excess serum GH/IGF-1 level differentially affected the three major brain tissue compartments. As shown in Figure 1B–1D, the nGMV and nWMV were significantly greater in the GH-PA group than in the NonFun-PA (p < 0.01, p < 0.0001) and HC groups (p < 0.0001, p < 0.0001). In contrast, the nCSFV in the GH-PA group was significantly lower than that in the other groups (p < 0.0001).

### The correlation between the three main tissue compartments and excess GH/IGF-1 production

**Figure 2:**
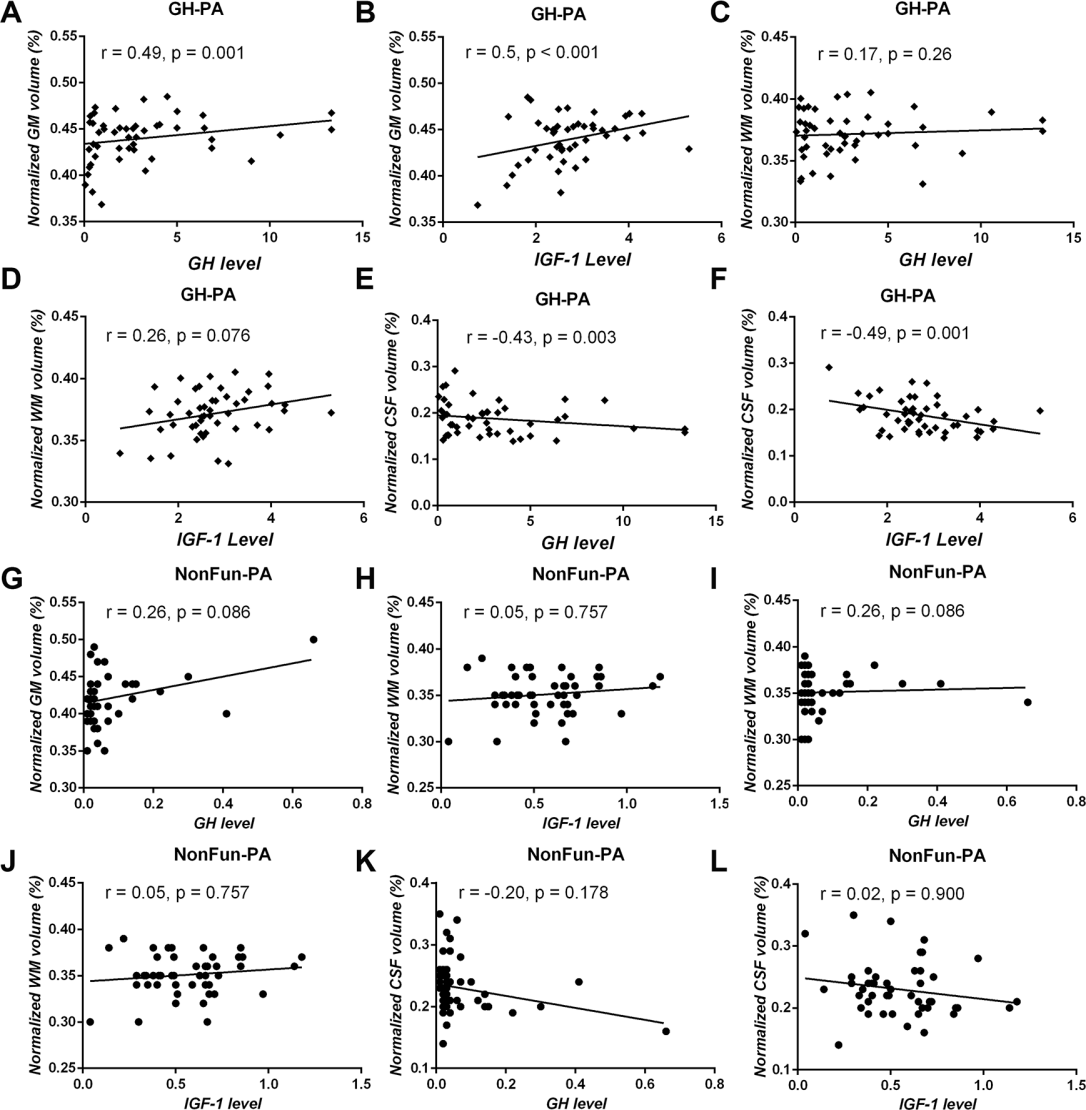
**Correlation analysis between serum GH/IGF-1 levels and brain tissue volume in patients with GH-PA and patients with NonFun-PA groups.** The normalized GM volume (nGMV) shows significant positive correlation with GH (**A**) and IGF-1 (**B**) in patients with GH-PA. The normalized WM volume (nGWV) shows no significant correlation with GH (**C**) or IGF-1 (**D**) in patients with GH-PA. The normalized CSF volume (nCSFV) shows significant negative correlation with GH (**E**) and IGF-1 (**F**) in patients with GH-PA. In patients with NonFun-PA, nGMV, nWMV, and nCSFV show no significant correlation with GH/IGF-1 (**G**–**L**).

To detect a relationship between excess GH/IGF-1 production and the three major brain tissue compartments in the GH-PA group, partial correlation analysis was applied. There was a significant positive correlation between GH/IGF-1 level and nGMV (Figure 2A, 2B; GH: r = 0.49, p = 0.001; IGF: r = 0.5, p < 0.001), but a strong negative correlation between GH/IGF-1 level and nCSFV in the GH-PA group (Figure 2E, 2F; GH: r = -0.43, p = 0.003; IGF: r = -0.49, p = 0.001). However, we observed no significant relationship between GH/IGF-1 level and nWMV in the GH-PA group (Figure 2C, 2D; GH: r = 0.17, p = 0.24; IGF: r = 0.28, p = 0.054). To detect the relationships between serum GH/IGF-1 levels and the three major brain tissue compartments in adults, partial correlation analysis was performed in the NonFun-PA group. No significant correlations between GH/IGF-1 and nGMV, nWMV, or nCSFV in Figure 2G–2L (p < 0.05). Furthermore, we performed partial correlation analysis between brain structures (nGMV, nWMV, nCSFV) and other hypophyseal hormones in the GH-PA group adjusting for age and sex. The results showed no significant relationships between other hypophyseal hormones and brain structures (nGMV, nWMV, nCSFV) in Table 2.

**Table 2:** Partial correlation analysis between hypophyseal hormones and brain structure (nGMV, nWMV, nCSFV) in GH-PA patients adjusting for age and sex.

**Correlation**	**nGMV**			**nWMV**			**nCSF**	
	**r-value**	**p-value**		**r-value**	**p-value**		**r-value**	**p-value**
Serum COR (ng/ml)	-0.264	0.076		0.006	0.967		0.176	0.241
Serum PRL (ng/ml)	0.040	0.794		0.052	0.732		-0.056	0.712
Serum LH (mIU/ml)	0.054	0.720		0.039	0.798		-0.053	0.724
Serum FSH (mIU/ml)	0.068	0.652		0.092	0.544		-0.095	0.529
Serum E2 (pg/ml)	0.199	0.184		0.022	0.887		-0.144	0.340
Serum P4 (ng/ml)	0.023	0.880		0.186	0.217		-0.120	0.425
Serum T (ng/ml)	-0.252	0.091		-0.189	0.207		0.282	0.058
Serum TSH (uTu/ml)	-0.073	0.630		-0.118	0.435		0.121	0.422

COR, cortisol; PRL, prolactin; LH, luteinizing hormone; FSH, follicle-stimulating hormone; E2, estradiol; P4, progesterone; T, testosterone; TSH, thyroid stimulating hormone.

### VBM analysis

### GM regional differences

**Figure 3:**
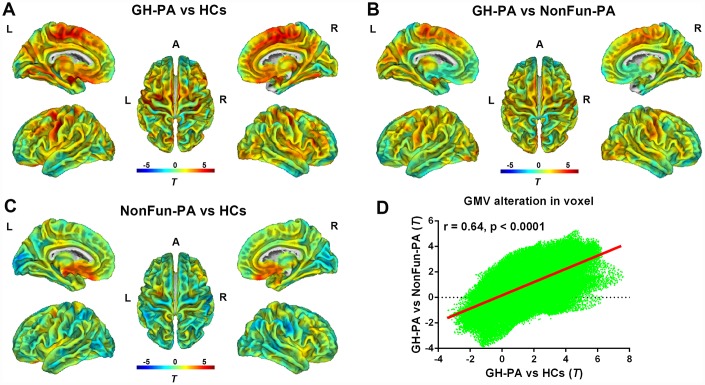
**Distribution of GMV alterations in voxel level among the three groups.** VBM analysis showing the GMV alteration in GH-PA vs HCs (**A**), in GH-PA vs NonFun-PA (**B**), in NonFun-PA vs HCs (**C**). Correlation analysis of GMV alteration pattern between GH-PA vs HCs and GH-PA vs NonFun-PA (**D**).

Other factors such as tumors and natural variations can affect brain structures. Therefore, we first performed whole-brain VBM analysis at the voxel level through the two-sample t-tests were performed, and found the differences in GMV between GH-PA and HCs groups, the GH-PA and NonFun-PA groups, and the NonFun-PA and HCs groups (Figure 3A–3C). Furthermore, we calculated the similarity of the GMV alteration pattern between the GH-PA and HCs groups and between GH-PA and NonFun-PA groups using Pearson’s correlation, and found a correlation coefficient of 0.64 (p < 0.0001, Figure 3D). VBM analysis showed extensive bilateral clusters of significantly increased GMV along the midline regions from the frontal cortex to parietal cortex, subcortical GM, and cerebellum GM in the GH-PA group compared with those in the other two groups (FDR-corrected, p < 0.05, Supplementary Figure 1A, 1B). No significant GMV alterations were observed in the cortex between NonFun-PA and HCs groups, except for in the regions close to the tumor. No cluster was identified to have a significantly lower GMV in the GH-PA group than in the controls.

### WM regional differences

**Figure 4:**
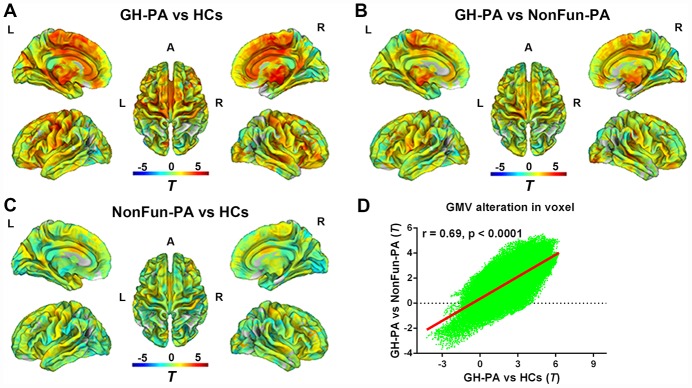
**Distribution of WMV alteration in voxel level among three groups.** VBM analysis showing the WMV alteration in GH-PA vs HCs (**A**), in GH-PA vs NonFun-PA (**B**), in NonFun-PA vs HCs (**C**). Correlation analysis of WMV alteration between GH-PA vs HCs and GH-PA vs NonFun-PA (**D**).

Similarly, Figure 4A–4C showed WMV alterations between the GH-PA and HCs groups, the GH-PA and NonFun-PA groups, and the NonFun-PA and HCs groups, respectively, using whole-brain VBM analysis at the voxel level. The WMV alteration pattern between the GH-PA and HCs groups and between the GH-PA and NonFun-PA groups showed a correlation coefficient of 0.69 (p < 0.0001, Figure 4D). VBM analysis showed revealed extensive bilateral clusters of significantly increased WMV in the entire brain, including the cerebellum and brain stem, in GH-PA compared to other two groups (Supplementary Figure 2A, 2B, FDR corrected, p < 0.05). There were no clusters with significant differences in WMV between the NonFun-PA and HC groups.

### RBM analysis

**Figure 5:**
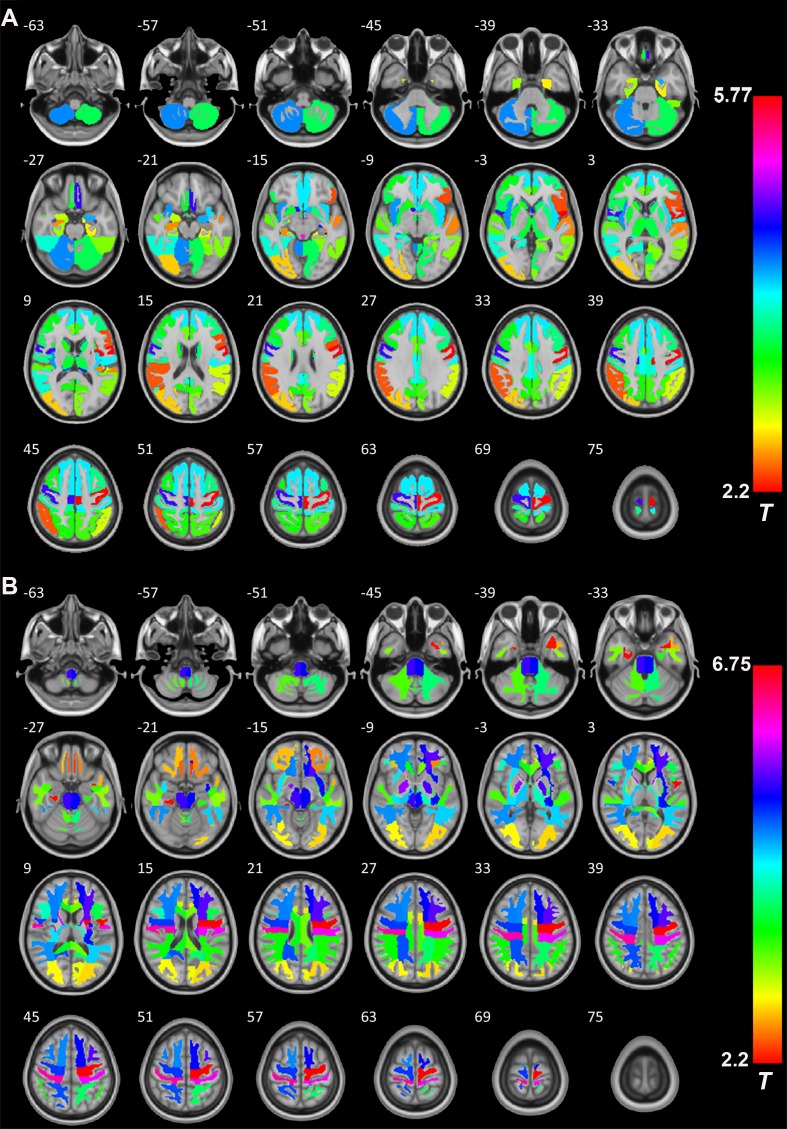
**The role of GH/IGF-1 in the GMV and WMV of brain regions.** (**A**) RBM analysis showing the increased GMV of 54 brain regions (Supplementary Table 1, from a total of 68 brain regions, Hammers' atlas) in patients with excess GH/IGF-1 production compared to that in HCs. (**B**) RBM analysis showing the white matter volume (WMV) of 54 brain regions (Supplementary Table 1, from a total of 68 brain regions, Hammers' atlas) increase in patients with excess GH/IGF-1 production compared to HCs. Significance was determined by uncorrected p < 0.05, using two-sample t-tests.

Next, we studied the detailed effects of excess serum GH/IGF-1 levels on the GMV and WMV in different brain regions by comparing the patients with GH-PA and HCs. RBM analysis was used to obtain the average volume of each brain region, according to Hammers’ atlas maps (a total of 68 brain regions). As shown in Figure 5A, the GMV of 54 brain regions (Supplementary Table 1) was significantly greater in the patients than in the HCs. As shown in Figure 5B, as assessment of the WM revealed that the WMV of 54 brain regions (Supplementary Table 1) was significantly greater in the patients than in the HCs.

### Relation of average GMV/WMV in the brain regions with excess GH/IGF-1 production

Furthermore, we performed correlation analyses between GH/IGF-1 levels and brain region volume based on the results of the RBM analysis. As shown in Supplementary Figure 3, the GMV of 18 brain regions (Supplementary Table 2) were significantly positively correlated with serum GH levels (p < 0.05). Meanwhile, the GMV of 19 brain regions (Supplementary Table 2) were significantly positively correlated with serum IGF-1 levels (p < 0.05). Regarding WM, as shown in Supplementary Figure 4, there were only 5 brain regions (Supplementary Table 3) was positively correlated with GH levels (p < 0.05), and 10 brain regions (Supplementary Table 3) was positively correlated with IGF-1 levels (p < 0.05).

## DISCUSSION

In the present study, the results showed the following: (a) Whole GMV and WMV are significantly increased at the expense of CSFV in patients with excess GH/IGF-1 production; (b) the increase in GMV and the reduction in CSFV were significantly correlated with serum GH/IGF-1 levels; (c) VBM showed that excess serum GH/IGF-1 levels can lead to widespread increases in GMV and WMV in patients with GH-PA; (d) at the regional level, the GMV and WMV of 54 brain regions (from a total of 68 brain regions) exhibited obvious increases due to excess serum GH/IGF-1 levels; (e) of all of the brain regions examined, the GMV in only a few of these brain regions showed striking correlations with GH/IGF-1 levels (GH, 18 brain regions; IGF-1, 19 brain regions). The findings from this study provide imaging evidence that GH and IGF-1 contribute to brain tissue growth in adult humans.

In our study, whole-brain analyses showed no TIV increase in patients with excess GH/IGF-1 levels, but significant increases in GMV and WMV at the expense of CSFV, as well as significant correlations between GH/IGF-1 and GMV/CSFV. The lack of a difference in TIV may have been because the groups were composed of age- and sex-match adults (20-68 years old) subjects, and the intracranial volume has been shown to be fixed since adolescence [30, 31]. Considering the potential effects of others hypophyseal hormones on human brain structures, partial correlation analysis was performed between brain structure and others hypophyseal hormones adjusting for age and sex [32, 33]. Our results showed no significant relationships between others hypophyseal hormone and brain structures, further demonstrating the definite effects of higher GH/IGF-1 levels on brain structure. The increase in brain tissue (GM, WM) in patients with excess GH/IGF-1 production may be attributed to the roles of GH/IGF-1 in stimulating protein synthesis and proliferation in neurons, glia, and oligodendrocytes, favoring neuronal survival, and inhibiting apoptosis as reported in animal studies and GH/IGF-1 are still vital regulators of brain structure in the postadolescence period [34]. In the patients with NonFun-PA, the lack of a significant relationship between GH/IGF-1 and brain structure may be due to the lower serum GH/IGF-1 level, and reduction of GH/IGF-1 receptor in the brain regions with increasing age. In humans and animals, aging is characterized by a progressive decrease in both GH/IGF-I levels and brain tissue (GM and white WM) volume and an increase in CSFV [11, 35, 36]. In our study, correlation analyses indicated that higher GH/IGF-1 levels were accompanied by a greater GMV and lower CSFV in GH-PA patients. These results suggest that maintaining the higher serum level of GH/IGF-1 may alleviate brain atrophy in aged humans.

Considering the effects of other factors, such as tumors and population characteristics, on brain structure, we calculated the similarity of the GMV alteration in voxels between GH-PA vs HCs and GH-PA vs NonFun-PA, and the results showed significant positive correlation. This finding indicates the universal effect of higher GH/IGF-1 levels on brain grey matter of population in voxel level. In our global GM analysis with corrected, VBM showed extensive bilateral clusters of significantly increased GMV along the midline regions from the frontal cortex to parietal cortex, subcortical GM, and cerebellum GM in patients with excess GH/IGF-1 production. The increase in volume in the extensive number of regions may be due to the expression of GH/IGF-1 receptors are modulated by higher GH/IGF-1 level [37]. The animal studies have reported that GH/IGF-1 receptors are throughout the entire brain, and mainly concentrated in the superficial and deep cortical layers, cerebellum, olfactory bulb, amygdala, thalamic nuclei, brain stem and hippocampus [4, 5]. We further study the effect of GH/IGF-1 on specific brain regions (Hammers’ atlas) with RBM analysis, the results showed that 54 brain regions from a total of 68 brain regions increased the GMV. These findings are generally in line with those of other studies which reported GH or IGF-1 gene knock-out mice showed certain areas of the brain are significantly smaller compared to wild-type controls [13, 14]. The non-affected regions (only 14 in total) included the bilateral anterior medial temporal lobe, anterior lateral temporal lobe, inferior middle temporal gyrus, fusiform gyrus, pallidum, corpus callosum, and orbitofrontal gyrus. One previous study have reported that distribution of lowest IGF-1 receptor density in pallidum and corpus callosum compared to others regions being studied [38]. We speculate that no significant increase of these regions volume may be due to lower distribution of GH/IGF-1 receptor. Taken together, our results provide clear evidence that higher GH/IGF-1 levels can lead to increase of grey matter volume in various brain regions in adult humans.

In neurodegenerative diseases, such as amyotrophic lateral sclerosis (ALS) and Alzheimer’s disease (AD), the role of GH/IGF-1 has also been reported. In animal studies, some researchers have suggested that GH/IGF-1 administration can decrease Ab1–40 protein levels, which may contribute to age-related cognitive deficits, and promote improvements in learning and memory in aged animals with AD [39]. Similarly, a few clinical studies have reported that a dramatic decline in serum GH/IGF-1 levels in patients with AD and subsequent IGF-1 administration could provide significant benefits to these patients [24, 40]. In patients affected by ALS, a significant reduction in GH/IGF-1 secretion was found, and the efficacy of GH/IGF-1 treatment in improving muscle force, motor coordination, and protection of motor neurons has been controversial in the clinic [41, 42]. Meanwhile, structural MRI analyses have shown multiple brain regions with decreased GMV, and there was a significant correlation between brain atrophy and neurological dysfunction in conditions such as ALS, AD, Parkinson’s disease, and prion disorders. For example, restricted regional GM atrophy mainly in the medial temporal structures, including the bilateral hippocampus, parahippocampal gyrus, amygdala and entorhinal cortex, as well as the posterior cingulate gyrus and medial thalamus, and reductions in WMV in the left parahippocampal gyrus extending to the temporal WM, right temporal WM extending to the parahippocampal gyrus and posterior corpus callosum, were found in patients with AD by VBM analysis [43, 44]. In patients with ALS, VBM analysis showed that diffuse GMV reduction in primary motor cortex bilaterally, frontotemporal areas, cerebellum and basal ganglia [45]. Interestingly, our results indicated that higher serum GH/IGF-1 level can markedly enhance the volume of these brain regions, such as the bilateral hippocampus, amygdalae, entorhinal cortex, thalamus, precentral gyrus, insula, and posterior cingulate gyrus, using structural MRI analysis in humans. Furthermore, the correlation test showed a significant positive association between GH/IGF-1 with some of these brain regions volume. These findings suggest that GH/IGF-1 should be considered as a potential preventive and therapeutic strategy for various neurodegenerative diseases, but its clinical application require further study.

GH/IGF-1 was shown to contribute to the proliferation of oligodendrocytes, axonal regeneration, and myelination in experimental studies [46–48]. In humans, diffusion tensor imaging (DTI) analysis showed WM recovery after traumatic brain injury (TBI) was greater in patients with higher serum IGF-I levels [49]. In our study, we observed there were significant correlation of WMV alteration between GH-PA vs HCs and GH-PA vs NonFun-PA, as well as significantly increased volumes in widespread brain WM regions in the patients with high levels of GH/IGF-1 using VBM and RBM analysis. However, the correlation analysis showed no significant relationship between nWMV and excess circulating GH/IGF-1 levels. The role of excess circulating GH/IGF-1 levels in axon and myelination need further study due to the WMV increase can’t reflect the detail alteration of axon and myelination.

In present study, there are some limitations. Firstly, lack of diffusion tensor imaging (DTI) data to analysis of the effect of excess circulating GH/IGF-1 levels on axonal structural integrity. Second, no longitudinal imaging data collected from patients with GH-secreting pituitary adenoma after surgery to study whether the decrease of serum level of GH/IGF-1 lead to the alteration of brain structure. Third, there are no neuropsychological and cognitive assessment in the subjects to investigate the effect of higher GH/IGF-1 on cognitive function.

## CONCLUSIONS

High serum GH/IGF-1 levels significantly increased GMV and WMV in most regions and strikingly correlated with brain structure in adult humans. Our results provide imaging evidence that serum GH/IGF-1 contributes to brain growth, indicating that GH/IGF-1 may be a potential treatment option for neurodegenerative disorders and brain injury in humans.

## MATERIALS AND METHODS

### Participants

Forty-eight patients with GH-PA, who accepted therapy in the department of neurosurgery, Beijing Tiantan Hospital, were enrolled. The inclusion criteria for the patient group were being 18–70 years of age and having a diagnosis of GH-PA, according to clinical manifestations, GH/IGF-1 serum levels, and postoperative pathological diagnosis. Forty-eight age- and sex-matched clinical NonFun-PA patients and forty-eight age- and sex-matched healthy controls (HCs) constituted the control groups. The exclusion criteria for the patient and control groups included a history of stroke, cerebral trauma, or other intracranial space-occupying lesions; neurodegenerative diseases; substance use disorders for alcohol or heroin; and an inability to complete the MRI examinations.

The Beijing Tiantan hospital ethics committee approval and informed written consent from the participants were obtained, in accordance with the Declaration of Helsinki. The relevant guidelines and regulations were strictly observed in our experimental procedures.

### Endocrine evaluation

All patients had completed endocrine testing (GH, IGF-1, cortisol, prolactin, luteinizing hormone, follicle-stimulating hormone, estradiol, progesterone, testosterone, thyroid stimulating hormone) for the study. Venous blood samples were collected between 06:00 a.m. and 10:00 a.m. following 10–12 h of fasting. The serum value of hormones were measured using the IMMULITE 2000 immunoassay system (Siemens). To remove the effect of age and sex, IGF-1 and GH levels were calculated as follows: serum IGF-1 or GH value/95^th^ percentile of the age- and sex-adjusted normal range [29].

### Image acquisition

All patients were scanned on the 3T Philips Ingenia CX MRI scanner (Philips Healthcare, Best, the Netherlands) using a commercial 32-channel head coil. The T1-weighted (T1W) images were obtained with a 3D sagittal magnetization-prepared rapid acquisition gradient echo (MPRAGE) sequence (resolution of 1 mm isotropic voxels; field of view = 240 × 240 mm^2^; 196 slices; flip angle = 8°; inversion time (TI)/repetition time (TR)/echo time (TE) = 494/1339/3.0 ms; data matrix = 240 × 240; bandwidth = 241 Hz/px; SENSE = 2). The structural images of all participants were examined for possible lesions in the cortex, as an exclusion criterion, by two expert radiologists.

### Data preprocessing and analysis

Image data processing was conducted using SPM12 (http://www.fil.ion.ucl.ac.uk/spm) and the CAT12 toolbox (http://www.neuro.uni-jena.de/cat/) in the MATLAB environment (MATHWORKS, California, USA). First, using the module “Segment Data” of CAT12, we segmented every T1W image into GM, WM, and CSF. The modulated warped GM images were then normalized to Montreal Neurological Institute (MNI)-152 standard space with an isotropic voxel resolution of 1.5 mm × 1.5 mm × 1.5 mm. Next, using “Display one slice for all images”, we checked the data quality to determine if some reasonable results were obtained after the segmentation and normalization procedures. Using a boxplot and correlation matrices, we also checked sample homogeneity to identify outliers by visualizing the correlation between the volumes. The modulated GM map of each individual was smoothed with an 8-mm full-width at half-maximum Gaussian kernel.

The absolute GM volume (GMV), absolute WM volume (WMV), and absolute CSF volume (CSFV) as well as total intracranial volume (TIV) of each subject were estimated using the “Estimate TIV” module. To correct for variation in subjects’ head sizes, we calculated the normalized GM volume (nGMV), normalized WM volume (nWMV), and normalized CSF volume (nCSFV) by dividing the individual subject’s GMV, WMV, and CSFV values by each subject’s respective TIV value. TIV, nGMV, nWMV, and nCSFV were compared among the three groups using one-way ANOVA with Bonferroni’s multiple comparisons test. The statistical analyses were performed using Statistical Package for Social Sciences software (SPSS, ver. 25.0; Chicago, IL) with p ˂ 0.05 as the significance level.

### Voxel-based morphometry (VBM) analysis

A two-sample t-test was applied in CAT12 to assess significant differences among the three groups with TIV, age, and sex as covariates. We calculated the similarity of the GMV/WMV alteration pattern between GH-PA and HCs and between GH-PA and NonFun-PA using Pearson’s correlation based on *T* values of the group level analysis in the whole brain. To further confirm the difference in GMV/WMV between two groups, significance was determined using positive false discovery rate (pFDR) < 0.05 at the voxel level and cluster sizes k > 333.

### Region-based morphometry (RBM) analysis

The averaged GMV, WMV and CSFV from each region were extracted according to the Hammers atlas (three-dimensional maximum probability atlas of the human brain, with particular reference to the temporal lobe; Hammers, 2003). The normalized volume of each brain region between patients and controls was compared using two-sample t-tests. The statistical analyses were performed using SPSS with p ˂ 0.05 as the significance level.

### Correlation analysis

Partial correlation analysis was performed to determine the correlation between the three main tissue compartments (nGMV, nWMV, nCSFV) and serum hormone levels (GH, IGF-1, cortisol, prolactin, luteinizing hormone, follicle-stimulating hormone, estradiol, progesterone, testosterone, thyroid stimulating hormone) in patients with GH-PA and NonFun-PA adjusting for age and sex. p < 0.05 was considered statistically significant, and analyses were conducted using SPSS.

## Supplementary Material

Supplementary Figures

Supplementary Tables
