# Correction for: The role of serum growth hormone and insulin-like growth factor-1 in adult humans brain morphology

**DOI:** 10.18632/aging.203601

**Published:** 2021-09-29

**Authors:** Taoyang Yuan, Jianyou Ying, Lu Jin, Chuzhong Li, Songbai Gui, Zhenye Li, Rui Wang, Zhentao Zuo, Yazhuo Zhang

**Affiliations:** 1 Beijing Neurosurgical Institute, Capital Medical University, Beijing, China; 2 Department of Neurosurgery, Beijing Tiantan Hospital, Capital Medical University, Beijing, China; 3 State Key Laboratory of Brain and Cognitive Science, Institute of Biophysics, Chinese Academy of Sciences, Beijing, China; 4 CAS Center for Excellence in Brain Science and Intelligence Technology, Chinese Academy of Sciences, Beijing, China; 5 Sino-Danish College, University of Chinese Academy of Sciences, Beijing, China; 6 Beijing Institute for Brain Disorders, Brain Tumour Center, China National Clinical Research Center for Neurological Diseases, Key Laboratory of Central Nervous System Injury Research, Beijing, China

**Keywords:** correction

Original article: Aging. 2020; 12:1377–1396.  . https://doi.org/10.18632/aging.102688

**This article has been corrected:** The authors recently found that panels **H** and **J** in **Figure 2** are the same, as they accidentally placed normalized WM plot instead of normalized GM plot. They replaced panel **H Figure 2** with the correct plot from the original data. This alteration does not affect the results or conclusions of this work.

New **Figure 2** is presented below.

**Figure 2:**
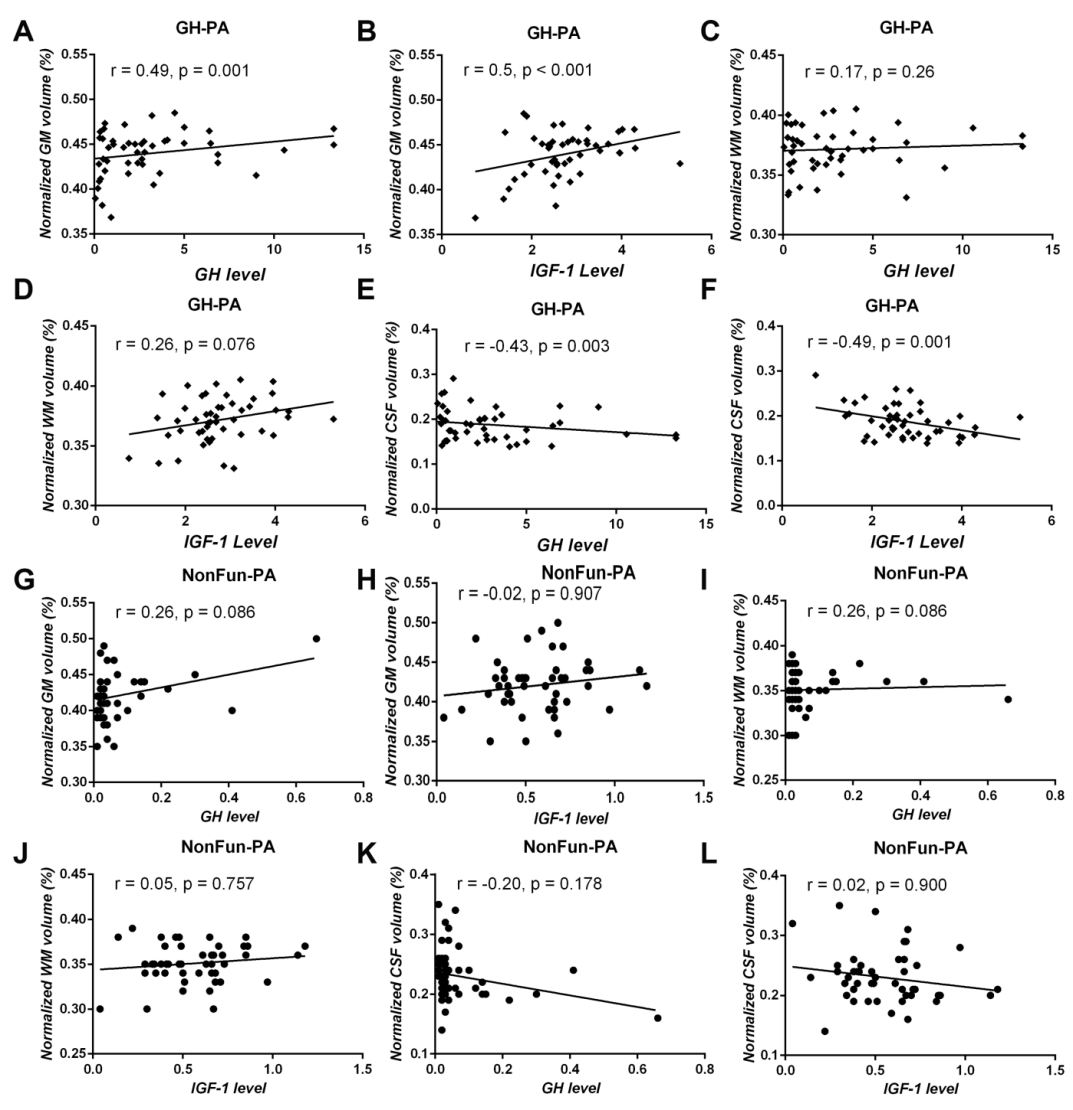
**Correlation analysis between serum GH/IGF-1 levels and brain tissue volume in patients with GH-PA and patients with NonFun-PA groups.** The normalized GM volume (nGMV) shows significant positive correlation with GH (**A**) and IGF-1 (**B**) in patients with GH-PA. The normalized WM volume (nGWV) shows no significant correlation with GH (**C**) or IGF-1 (**D**) in patients with GH-PA. The normalized CSF volume (nCSFV) shows significant negative correlation with GH (**E**) and IGF-1 (**F**) in patients with GH-PA. In patients with NonFun-PA, nGMV, nWMV, and nCSFV show no significant correlation with GH/IGF-1 (**G–L**).

