# Supplementary material for: The role of serum growth hormone and insulin-like growth factor-1 in adult humans brain morphology
Source: Aging (Albany NY). 2020 Jan 22;12(2):1377–96. doi: 10.18632/aging.102688 (PMC7053622; doi:10.18632/aging.102688)
Supplement: Supplementary Figures [file aging-12-102688-s002..pdf]

SUPPLEMENTARY FIGURES

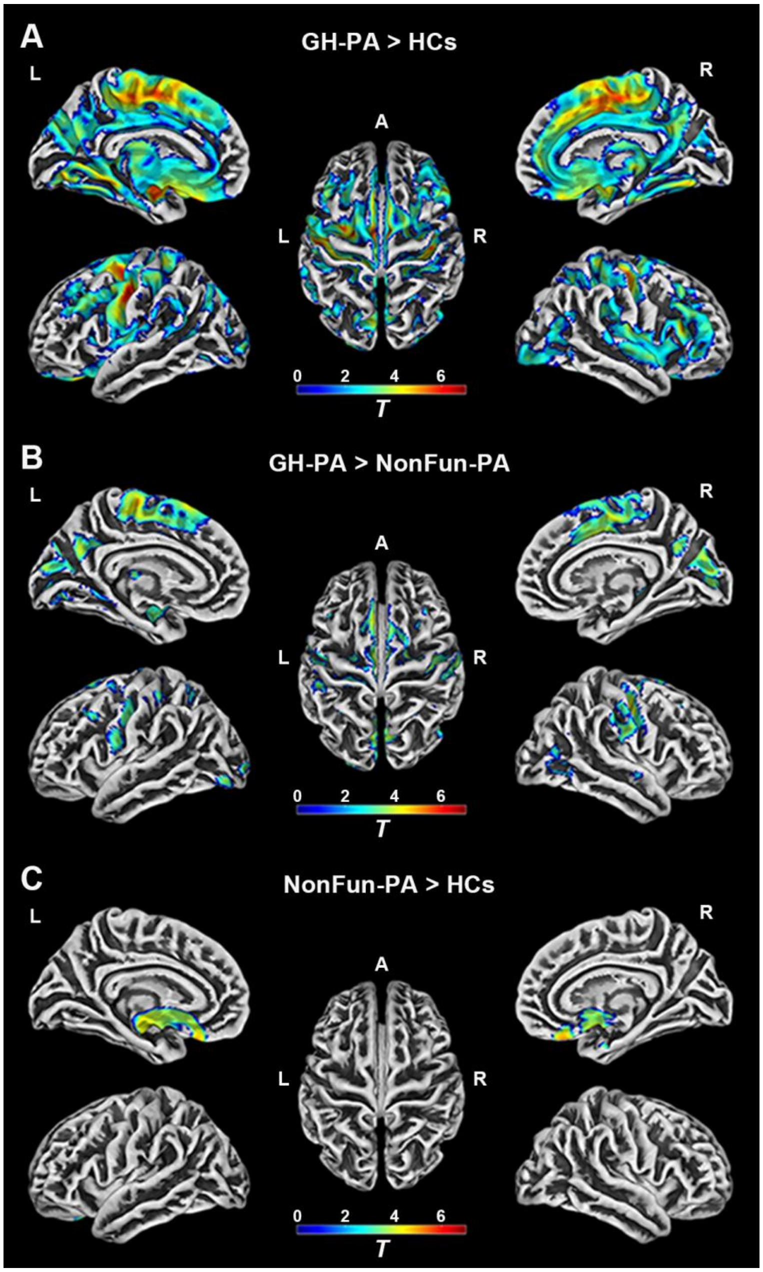

Supplementary Figure 1. GM regional differences. VBM analysis showing the extensive regions of GMV increases in GH-PA compared to HCs (**A**) and NonFun-PA (**B**) (FDR corrected, $p < 0.05$, cluster size>333). VBM analysis showing GMV alteration in NonFun-PA compared to HCs (**C**) (FDR corrected, $p < 0.05$, cluster size>333).

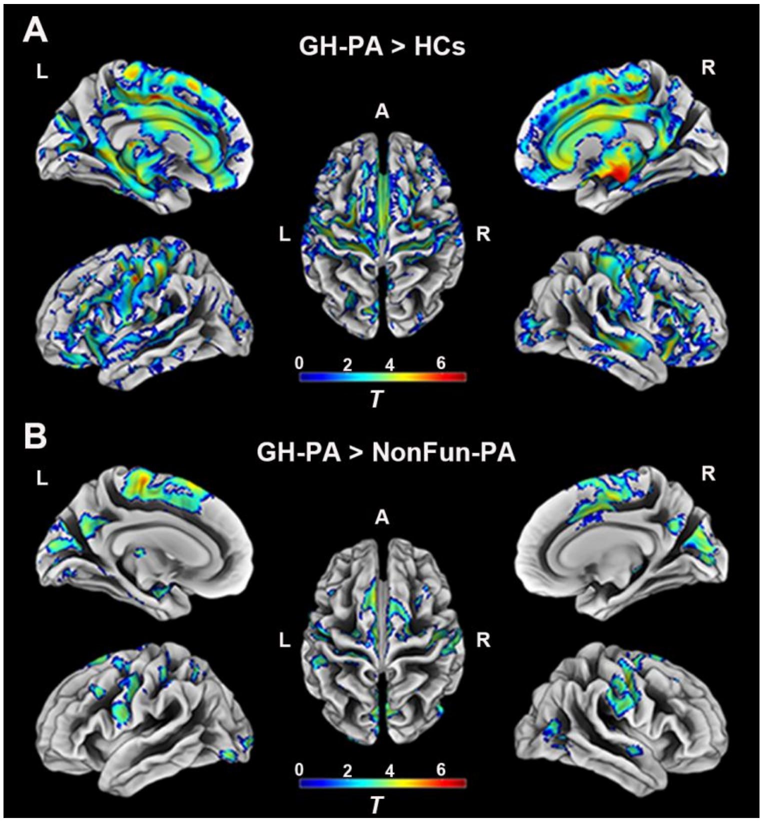

Supplementary Figure 2. WM regional differences. VBM analysis showing the extensive regions of WMV increases in GH-PA compared to HCs (**A**) and NonFun-PA (**B**) (FDR corrected, p < 0.05, cluster size>333).

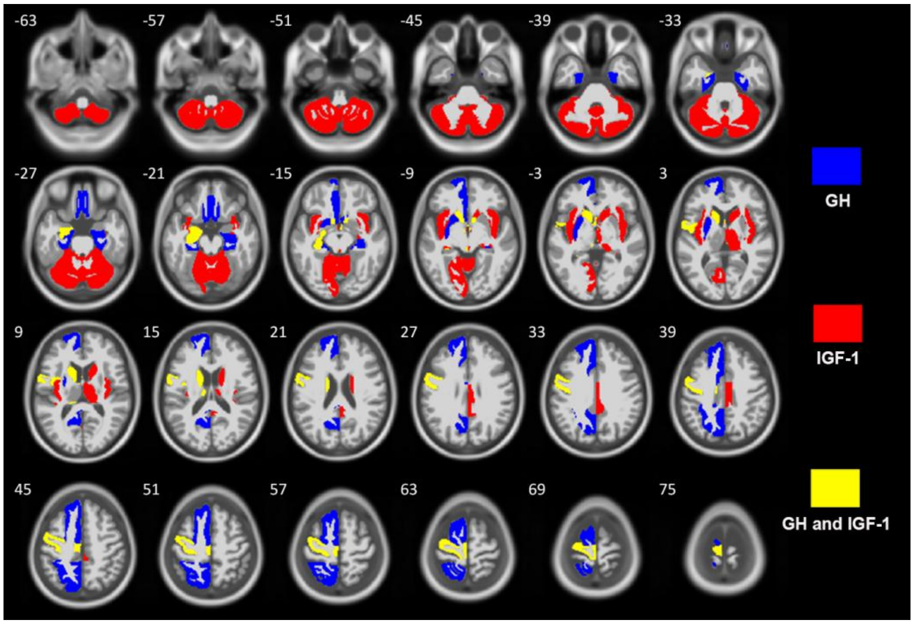

Supplementary Figure 3. Correlation analysis between GH/IGF-1 and gray matter volume (GMV) of brain regions (Hammers' atlas) in GH-PA patients. Blue represents brain regions volumes that are positively correlated with serum GH levels; red represents brain region volumes that are positively correlateed with serum IGF-1 levels; yellow represents brain region volumes that are simultaneously positively correlated with serum GH levels and IGF-1 levels (Supplementary Table 2). Significance was determined by p < 0.05.

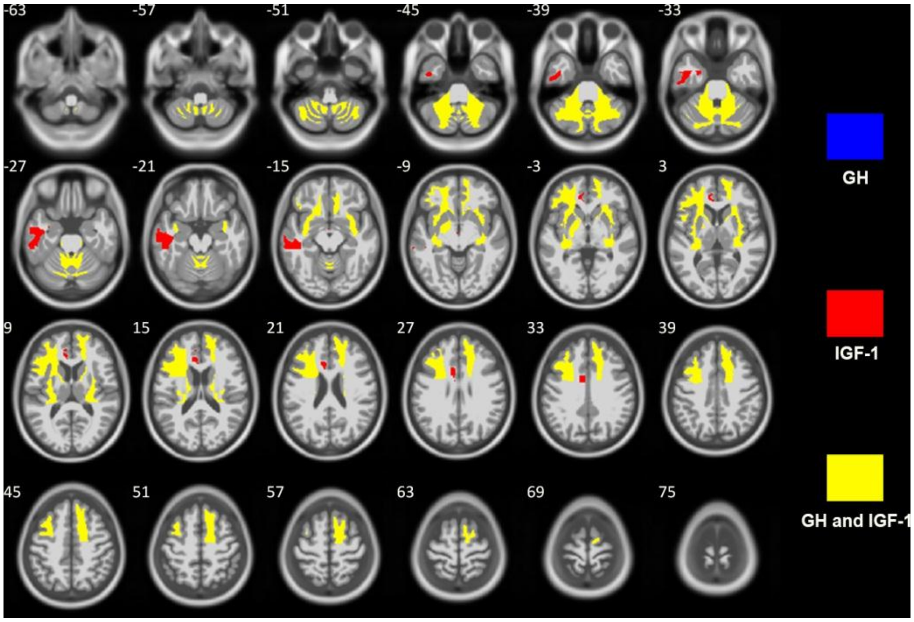

Supplementary Figure 4. Correlation analysis between GH/IGF-1 and white matter volume (WMV) of brain regions (Hammers' atlas) in GH-PA patients. Blue represents brain region volumes that are positively correlated with serum GH levels; red represents brain regions volumes that are positively correlated with serum IGF-1 levels; yellow represents brain region volumes that are simultaneously positively correlated with serum GH levels and IGF-1 levels (Supplementary Table 3). Significance was determined by $p < 0.05$.
