# Supplementary material for: The role of serum growth hormone and insulin-like growth factor-1 in adult humans brain morphology
Source: Aging (Albany NY). 2020 Jan 22;12(2):1377–96. doi: 10.18632/aging.102688 (PMC7053622; doi:10.18632/aging.102688)
Supplement: Supplementary Tables [file aging-12-102688-s001..pdf]

SUPPLEMENTARY TABLES

Supplementary Table 1. Specific brain regions of gray matter (GM) volume and white matter (WM) volume affected by serum GH/IGF-1 level in patients compared with controls (Hammers' atlas).

Abnormal brain region (GM)	T value	Abnormal brain region (WM)	T value
lHip	2.10	rHip	2.11
rHip	2.52	lAntMedTeLo	2.26
lAmy	4.17	lAntLatTeLo	2.73
rAmy	2.95	lAmbParHipGy	3.64
lAmbParHipGy	2.76	rAmbParHipGy	2.25
rAmbParHipGy	3.02	lSupTemGy	3.57
lSupTemGy	2.51	rSupTemGy	3.51
rSupTemGy	3.20	lInfMidTemGy	3.30
lCbe	3.58	rInfMidTemGy	3.37
rCbe	4.26	lCbe	4.05
lBst	5.16	rCbe	3.48
rBst	5.22	lBst	5.25
lIns	4.06	rBst	5.40
rIns	4.23	lIns	5.19
lLatOcLo	2.03	rIns	4.55
rLatOcLo	2.68	lLatOcLo	2.84
lAntCinGy	3.30	rLatOcLo	2.95
rAntCinGy	3.12	lAntCinGy	2.89
lPosCinGy	3.83	rAntCinGy	3.15
rPosCinGy	3.97	lPosCinGy	3.55
lMidFroGy	3.68	rPosCinGy	3.74
rMidFroGy	3.45	lMidFroGy	5.49
lPosTeLo	3.10	rMidFroGy	4.86
rPosTeLo	3.88	lPosTeLo	4.61
lInfLatPaLo	2.90	rPosTeLo	4.81
rInfLatPaLo	2.40	lInfLatPaLo	3.67
lCauNuc	3.60	rInfLatPaLo	3.72
rCauNuc	3.32	lCauNuc	5.64
lAccNuc	4.15	rCauNuc	5.83
rAccNuc	4.70	lPut	3.79
lPut	3.66	rPut	3.31
rPut	3.49	lTha	4.26
lTha	3.55	rTha	4.64
rTha	3.50	lPal	5.43
lLatTemVen	4.51	rPal	5.69
rLatTemVen	4.36	lCC	3.39
l3thVen	4.02	rCC	3.78
r3thVen	3.69	lLatTemVen	4.01
lPrcGy	5.77	rLatTemVen	3.93

rPrcGy	4.73	lPrcGy	6.75
lRecGy	4.71	rPrcGy	5.07
rRecGy	3.51	lRecGy	2.39
lInfFroGy	2.38	rRecGy	2.49
rInfFroGy	3.60	lOrbFroGy	2.60
lSupFroGy	4.02	rOrbFroGy	2.77
rSupFroGy	4.02	lInfFroGy	3.93
lPoCGy	4.01	rInfFroGy	4.26
rPoCGy	3.73	lSupFroGy	5.25
lSupParGy	3.26	rSupFroGy	4.82
rSupParGy	3.36	lPoCGy	5.94
lLinGy	3.61	rPoCGy	6.24
rLinGy	2.06	lSupParGy	4.01
lCun	3.10	rSupParGy	5.00
rCun	2.07	lCun	3.02

Supplementary Table 2. Specific gray matter (GM) regions correlated with serum GH/IGF-1 level in patients (Hammers' atlas).

GH Correlation	r value	p-value	IGF-1 Correlation	r value	p value
Grey Matter			Grey Matter		
lHip	0.42	<0.05	lHip	0.36	<0.05
rHip	0.31	<0.05	lAmy	0.37	<0.05
lAmy	0.43	<0.05	lCbe	0.32	<0.05
lAmbParHipGy	0.43	<0.05	rCbe	0.31	<0.05
rAmbParHipGy	0.42	<0.05	lBst	0.43	<0.05
lBst	0.34	<0.05	rBst	0.37	<0.05
lCauNuc	0.29	<0.05	lIns	0.34	<0.05
lAccNuc	0.64	<0.05	rIns	0.36	<0.05
rAccNuc	0.62	<0.05	rPosCinGy	0.30	<0.05
lPut	0.38	<0.05	lCauNuc	0.43	<0.05
lCC	0.32	<0.05	rCauNuc	0.37	<0.05
lLatTemVen	0.30	<0.05	lAccNuc	0.34	<0.05
r3thVen	0.37	<0.05	rAccNuc	0.39	<0.05
lPrcGy	0.32	<0.05	rTha	0.35	<0.05
lRecGy	0.44	<0.05	lLatTemVen	0.31	<0.05
rRecGy	0.42	<0.05	rLatTemVen	0.32	<0.05
lSupFroGy	0.29	<0.05	l3thVen	0.36	<0.05
rSupFroGy	0.32	<0.05	r3thVen	0.41	<0.05
			lPrcGy	0.35	<0.05

Supplementary Table 3. Specific white matter (WM) regions correlated with serum GH/IGF-1 level in patients (Hammers' atlas).

GH Correlation	r value	p value	IGF-1 Correlation	r value	p value
White Matter			White Matter		
lAmy	0.36	<0.05	lCbe	0.32	<0.05
lInfMidTemGy	0.32	<0.05	rCbe	0.37	<0.05
lAntCinGy	0.30	<0.05	lIns	0.32	<0.05
l3thVen	0.33	<0.05	rIns	0.29	<0.05
rPrcGy	0.32	<0.05	lMidFroGy	0.32	<0.05
			lCauNuc	0.30	<0.05
			rCauNuc	0.29	<0.05
			lPal	0.32	<0.05
			lInfFroGy	0.29	<0.05
			rSupFroGy	0.29	<0.05
